# Operationalizing the RE-AIM framework to evaluate the impact of multi-sector partnerships

**DOI:** 10.1186/1748-5908-9-74

**Published:** 2014-06-12

**Authors:** Shane N Sweet, Kathleen A Martin Ginis, Paul A Estabrooks, Amy E Latimer-Cheung

**Affiliations:** 1 Department of Kinesiology and Physical Education, McGill University, 475 Pine Ave W, Montreal, Quebec H2W 1S4, Canada; 2 School of Kinesiology and Health Studies, Queen’s University, 28 Division St, Kingston, Ontario K7L 3N6, Canada; 3 Department of Kinesiology, McMaster University, 1280 Main Street West, Hamilton, Ontario L8S 4K1, Canada; 4 Department of Human Nutrition, Food and Exercise and Carilion Clinic, 1 Riverside Circle, Suite 104, Roanoke, Virginia 24016, USA

**Keywords:** RE-AIM, Partnerships, Spinal cord injury, Physical activity

## Abstract

**Background:**

The RE-AIM (Reach, Effectiveness, Adoption, Implementation, and Maintenance) framework is a reliable tool for the translation of research to practice. This framework has been widely applied to assess the impact of individual interventions. However, RE-AIM has rarely been used to evaluate implementation interventions, especially from multi-sector partnerships. The primary purpose of this paper is to operationalize the RE-AIM approach to evaluate large, multi-sector partnerships. SCI Action Canada, a community-university partnership aimed to promote physical activity among adults with spinal cord injury, is used as an example. A secondary purpose is to provide initial data from SCI Action Canada by using this conceptualization of RE-AIM.

**Methods:**

Each RE-AIM element is operationalized for multi-sector partnerships. Specific to SCI Action Canada, seven reach calculations, four adoption rates, four effectiveness outcomes, one implementation, one organizational maintenance, and two individual maintenance outcomes are defined. The specific numerators based on SCI Action Canada activities are also listed for each of these calculations.

**Results:**

The results are derived from SCI Action Canada activities. SCI Action Canada’s reach ranged from 3% (end-user direct national reach) to 37% (total regional reach). Adoption rates were 15% (provincial level adoption) to 76% (regional level adoption). Implementation and organizational maintenance rates were 92% and 100%, respectively.

**Conclusions:**

We have operationalized the RE-AIM framework for larger multi-sectoral partnerships and demonstrated its applicability to such partnerships with SCI Action Canada. Future partnerships could use RE-AIM to assess their public health impact.

## Background

The RE-AIM framework is a valuable tool for implementation scientists, health promotion professionals and practitioners. It can be applied to assist with the translation of research to practice and to estimate the public health impact of programs and interventions [1-3]. RE-AIM is an acronym for the framework’s five evaluation components: Reach, Effectiveness, Adoption, Implementation, and Maintenance. Through these components, the impact of innovations can be assessed at both the individual (*i.e*., end-user) and organizational (*i.e*., delivery agent) levels.

Specifically, RE-AIM provides a functional starting point for determining the public health impact of strategies involved in health promotion by guiding the assessment of: reach, which captures the percentage of people from a given population who participate in a program and describes their characteristics; effectiveness, which refers to the positive and negative outcomes of the program; adoption*,* which is generally defined as the percent of possible settings (*e.g*., organizations) and staff that have agreed to participate in the program; implementation, which is an indicator of the extent to which the program was delivered as intended and its cost; and maintenance, which, at the individual level, reflects maintenance of the primary outcomes (>6 months). At the organizational level, maintenance captures the sustainability of the delivered programs.

RE-AIM has been applied to evaluate intervention impact in a variety of settings and across a broad range of behavioral outcomes [3,4], including weight loss [5,6], nutrition [7], injury prevention [8,9] and physical activity [7,10]. Most of these applications have focused on examining the effectiveness of a single intervention in achieving behavior change at the level of a patient, community member, student or employee [11]. To a lesser degree, the RE-AIM framework has been applied to understand the impact of implementation interventions – those that focus on the organizations and staff who are intended to implement clinical practice guidelines or to deliver evidence-based interventions with fidelity [12]. The framework has been used to evaluate the impact of an individual intervention within the context of a broader implementation intervention [13] and recently to assess broader multifaceted implementation interventions [14].

While each of the above RE-AIM applications provide valuable information on the impact of individual evidence-based interventions and training programs, there are limited reports of the impact – across all five RE-AIM dimensions – of broad, multi-faceted initiatives that incorporate multiple interventions targeted to a variety of audiences (*e.g*., healthcare providers, health educators, and other key stakeholders across sectors and organizations). Finch and Donaldson [15] highlighted that adapting RE-AIM may be necessary for the multilevel nature of implementation type interventions in order to gain a better understanding of the implementation issues that arise from evaluating these interventions. This understanding will help us to further develop and properly evaluate real-world interventions [15]. Similar to the multifaceted nature of sport injury prevention interventions highlighted by Finch and Donaldson [15], a number of concurrent interventions and approaches may be used in health promotion to achieve different goals and different RE-AIM benchmarks (*e.g*., reach versus implementation). For instance, some activities may be designed to reach as many people as possible (*e.g*., awareness campaigns), whereas others might be designed with specific implementation or maintenance goals in mind (*e.g*., quality improvement strategies). Because different activities would address different RE-AIM dimensions, in order to obtain a complete picture of the program’s overall impact, it would be necessary to conduct a RE-AIM evaluation across the program’s full range of activities. The RE-AIM framework has not yet been applied in this way to health promotion initiatives. Given increasing recognition of the value of multi-sector and partnered approaches to health promotion (*e.g*., [16,17]), there is a need for implementation scientists to have an established framework to evaluate the multiple, complementary activities that such approaches would entail. RE-AIM can serve as this framework as it has been deemed feasible to use in multi-sector partnerships [14,15,18]. The purpose of the present study was to examine the utility of RE-AIM for evaluating the impact of physical activity promoting activities conducted by a large, multi-sector partnership.

The evaluated partnership was SCI Action Canada (http://www.sciactioncanada.ca), a community-university partnership established in 2007 to ‘develop and mobilize strategies that will inform, teach and enable people living with spinal cord injury to initiate and maintain a physically active lifestyle’ [19]. Given that people with spinal cord injury (SCI) are at increased risk for developing chronic health conditions associated with a sedentary lifestyle [20], coupled with the finding that 50% of people with SCI participate in no leisure time physical activity whatsoever [21], physical activity promotion is a significant health issue for this population. It is a complex issue, however, that cannot be fully addressed by any one entity or organization. Rather, effective promotion requires a combination of academic and community-based expertise and involvement from a variety of disciplines and stake-holders [19]. As such, SCI Action Canada established multi-sectoral partnerships (*e.g*., with government agencies, non-governmental organizations, community groups) that have collaborated to develop evidence-based, physical activity-enhancing programs and initiatives (*e.g*., telephone-based physical activity counselling program, exercise videos and manuals) and resources (*e.g*., physical activity guidelines and Get Fit Toolkit) disseminated through a variety of formats (*e.g*., online, paper, verbal) and platforms (*e.g*., website, seminars, one-on-one).

When considering the evaluation of complex, multilevel health initiatives like SCI Action Canada, Mercer and colleagues [22] suggest that there are necessary trade-offs related to study design, measurement decisions, and outcomes that should be assessed. The result is a pragmatic approach that balances scientific control with practice-based implementation to include the adaptation of SCI Action Canada’s programs, initiatives and resources to the typical practice partner settings where individuals with SCI receive services [22,23]. Specifically, SCI Action Canada has taken an implementation-effectiveness-based approach rather an efficacy approach when evaluating its programs, initiatives and resources. Briefly, an efficacy approach is one that is highly controlled to maximize the likelihood that large effects are found, such as the methodological approach used in randomized controlled trials. An effectiveness approach differs insofar as there is little to no experimental control and the goal is to determine if the approach could be implemented successfully within typical community, clinical, or public health organizations [8]. Therefore, the SCI Action Canada partnership took a pragmatic approach to the application of the RE-AIM framework, rather than full employment of the model [4], to use data that is available, provides little burden on participants and staff that provide services, and provides information that can inform the future sustainability of the partnerships activities [23].

### Applying RE-AIM to evaluate a community-university partnership

When applying RE-AIM to evaluate the impact of a multi-sectoral partnership such as SCI Action Canada, there are three general approaches that could be taken. First, each activity undertaken by the partnership could be evaluated based on the RE-AIM dimension it targeted (*i.e*., activity-specific approach). For instance, awareness-raising strategies would target reach or adoption, physical activity promotion strategies might be used to improve effectiveness and maintenance, and provider-training strategies could be identified as methods to improve adoption and implementation of evidence-based guidelines. Each of these strategies could then be evaluated based on the degree to which the strategy reached its intended audience, was effective (*i.e*., in improving awareness, changing physical activity, or implementation of guidelines), could be broadly adopted across regions and implemented and sustained with fidelity. Yet, while this approach would provide excellent information on the impact of each strategy, it would be burdensome to implement and likely not feasible in most practice settings. Further, the summary of findings across strategies would be complex and not necessarily related to the overall impact of the partnership.

A second option would be an additive approach, whereby information collected from across the initiatives is amalgamated to inform each RE-AIM dimension. For example, the number of participants enrolled in all strategies to promote physical activity, the number of materials delivered to people with SCI, and the number of unique visitors to the partnership’s website could be summed to provide an indication of the reach of the partnership. Likewise, average effect sizes could be calculated across interventions to calculate mean effectiveness of the strategies. The drawback of this approach, however, is that it provides only a broad, overarching sense of impact and does not allow for determination of inter-relationships between RE-AIM dimensions (*e.g*., correspondence between the number of people reached for a particular physical activity promotion strategy and the effect size for that strategy).

A third option is a hybrid approach that is guided by pragmatic decisions [24] regarding which strategies deserve more thorough RE-AIM evaluation and which are best evaluated using one or two RE-AIM dimensions. As noted by Glasgow [23], pragmatic approaches focus on the perspectives of the stakeholders and can speed the integration of research, policy and practice. Intuitively, these approaches also reduce burden on participants and partner organizations to glean information that is actionable within a given context or across a range of contexts. Pragmatic approaches also focus on utilizing the strengths of different methods/perspectives that benefit the evaluation of a program (or partnerships such as SCI Action Canada) instead of strictly following one method/perspective [24]. Oliver [24] used the example of the debate between using quantitative or qualitative methodologies to evaluate learning technologies. In that example, the researcher would select the quantitative and/or qualitative approach that is most appropriate to the specific component being examined within the broader evaluation of the learning technologies. Similarly, we adopted a hybrid approach in this manuscript because it was more pragmatic to evaluate some RE-AIM elements with the activity-specific approach (*e.g*., effectiveness) while using an additive approach to assess other RE-AIM elements (*e.g*., reach). The hybrid approach is also ideal because it allows some of the scientific controls to be relaxed given that SCI Action Canada’s programs/initiatives/resources are delivered in the context of the ‘real-world’.

With these various options in mind, the primary purpose of this paper was to describe the development and application of a RE-AIM approach to evaluate a large, multi-sector partnership: SCI Action Canada. A secondary goal was to provide initial RE-AIM evaluation data for the SCI Action Canada partnership.

## Methods

### Partnership overview

SCI Action Canada (http://www.sciactioncanada.ca) was established in 2007, with a mission to ‘develop and mobilize strategies to inform, teach and enable people living with SCI to initiate and maintain a physically active lifestyle’ [19]. Formal community partners are based in the provinces of Ontario and Alberta and include 15 community organizations and 1 law firm. The university partners are represented by 15 researchers working in nine universities across two countries. To achieve its mission, SCI Action Canada has engaged the expertise of community and university partners throughout the knowledge to action cycle [25]. Examples of partnership-led initiatives include the development of evidence-based physical activity guidelines for adults with SCI [26], an informational toolkit [27], telephone-based counseling and peer-mediated home-base physical activity program [28], and a province-wide, community-based knowledge mobilization event to further promote these resources [29].

### Reach

Large partnerships with national distribution programs as well as localized initiatives and research programs – such as SCI Action Canada – warrant multiple indicators to capture an accurate picture of their reach. Furthermore, given the broad range of activities used by the partnership to reach the SCI community, audiences could be comprised of both the targeted end-users (*i.e*., adults with SCI) as well as key support persons/intermediaries, such as healthcare professionals and family members. Accordingly, our reach indices need to document contact with these different audiences and reflect whether the reach to the target end-user (*i.e*., adults with SCI) is direct or indirect through the key intermediaries. As such, we conceptualized three general categories of reach – indirect, intended and direct. Regardless of the category of reach, the targeted end-users are adults with SCI as this is consistent with the mission of SCI Action Canada. Thus, the reach categories reflect the path by which adults with SCI were exposed to SCI Action Canada’s programs, initiatives and resources (*i.e*., indirect, intended or direct). Each of these categories was constituted from different reach data sources and are used to calculate three levels of reach: national, regional and engaged.

First, indirect reach was defined as the number of Canadians without a SCI (*e.g*., family member, healthcare provider) who: were sent information from SCI Action Canada; were invited to a presentation; or, who participated in an initiative. We acknowledge that this reach index could be interpreted as an indicator of adoption because family members and, in particular, healthcare providers make decisions whether or not to provide these materials to their family or patients. However, the data described below do not provide details on the use of information gathered by family or healthcare professionals, and our assumption was that Canadians without a SCI serve as intermediaries who would redirect SCI Action Canada information to at least one Canadian with SCI. Because we are unable to know which website visitors had an SCI, these data were also included in the indirect reach method, so as not to inflate the other more direct reach indicators described below. The two sources of indirect reach data were:

1. Data source 1: the number of Canadians without a SCI who were invited to SCI Action Canada presentations, participated in an initiative or were sent SCI Action Canada resources (*e.g*., physical activity guidelines for adults with SCI);

2. Data source 2: unique Canadian visitors to the SCI Action Canada website.

Second, intended reach was defined as the number of resources that are sent out to SCI- or disability-specific organizations with the intended distribution to adults with SCI (*i.e*., end-user). Based on our discussions with these organizations and our experience, we made the assumption that the resources given to these organizations would be more likely to reach people with SCI than would resources delivered through the indirect reach channels. Intended reach was calculated from one data source:

1. Data source 3: the number of resources given to organizations (*e.g*., Spinal Cord Injury Ontario) that would be destined to Canadians with SCI.

Third, direct reach was defined as the number of Canadians with SCI who had direct contact with SCI Action Canada. Direct reach was calculated with three data sources:

1. Data source 4: the number of Canadians with SCI who attended SCI Action Canada events;

2. Data source 5: the number of Canadians with SCI who participated in SCI Action Canada programs, initiatives and research studies;

3. Data source 6: the number of resources given directly to Canadians with SCI.

Using these six data sources, reach was computed at the national, regional and engaged levels. At each level, reach consisted of the percentage of participants who were exposed to the various SCI Action Canada programs, initiatives and resources. Therefore, each reach calculation consisted of a numerator and a denominator. Because the target end-user group is Canadians with SCI, the denominator for all national reach indices was the total number of Canadians living with SCI (N = 85,556; [30]). Numerators for national reach were computed as follows:

1. Total National Reach. This numerator includes all individuals tabulated from the three categories of reach – indirect, intended and direct – and includes all six data sources.

2. End-user Intended and Direct National Reach. The second reach calculation is defined by intended and direct reach. The numerator for this reach calculation consisted of Data sources 3, 4, 5, 6.

3. End-user Direct National Reach. This reach assessment has the most conservative numerator as it only includes Canadians with SCI who had direct contact with SCI Action Canada (Data sources 4, 5, 6).

Three regional level reach values were calculated using the numerators described above, but counting only individuals from the two provinces that have community partners linked with SCI Action Canada, namely Ontario and Alberta. The denominator for the regional reach level was estimated at 42,607 adults with SCI living in Ontario and Alberta.

Reach at the engaged level was defined as the extent to which individuals who were invited to events/projects attended or participated in the events/projects. For SCI Action Canada, the ratio was computed with data based on research participation due to the availability of the data regarding project invitation. Therefore, the engaged level numerator was the actual number of Canadians with SCI who participated in an SCI Action Canada research project (Data source 5). The denominator for this reach indicator included Canadians with SCI who were contacted to participate in a SCI Action Canada research project (N = 1,472).

### Adoption

For assessing partnership adoption, the number of partners who were engaged in the partnership was used as the indicator. For SCI Action Canada, adoption was calculated at national, regional and engaged levels, as well as a provincial level in order to give a geographical perspective of our activities. For the national and regional adoption indicators, only one adoption data source was used - number of SCI Action Canada partners (N = 16). This number was the numerator for both the national and regional adoption levels. The adoption denominator at the national level represented all possible Canadian community organizations that provide services to Canadians with SCI and thus, could potentially be a partner with SCI Action Canada (N = 35 organizations). At the regional level, the adoption denominator represents all potential community partner organizations that are in Ontario and Alberta (*i.e*., the two provinces which SCI Action Canada has formal partnership), plus national level organizations that do not have provincial units (*n* = 21 organizations). At the engaged level, the numerator consisted of the number of community partners that have adopted a program, and the denominator was the number of SCI Action Canada partners. For the provincial level indicator, the number of provinces/territories that have community partnerships with SCI Action Canada was the numerator, and the denominator was the 13 provinces/territories of Canada.

### Implementation and organization maintenance

Our pragmatic approach to understanding implementation and organizational maintenance focused on the evaluation needs and resources of our key stakeholders. We considered collecting specific implementation information for each of the SCI Action Canada activities across regions and provinces. However, this approach would require a large commitment of stakeholder resources and would split their focus between implementation and implementation-evaluation. There was some concern that pushing for the most rigorous evaluation possible would run the risk of compromising the partner’s sense of ownership of the evaluation outcomes and process as well as stretch available resources too broadly.

After careful consideration of these issues and conversations with our partners, we determined that the most informative implementation and sustainability indicators that could be monitored were the degrees to which the partnership’s strategic plan was completed as intended. Although we recognize that by taking this approach we relegated some scientific control over the implementation activities and evaluation, a benefit of using the partnership’s strategic plan is that this approach is more in line with the goal of evaluating the partnership as a whole rather than the sum of all its programs/initiatives/resources. Thus, we conceptualized implementation based on the number of projects in the partnership’s strategic plan. The 25 programs/initiatives/resources implemented between 2007 and 2013 serve as the implementation denominator (a complete list of programs/initiatives/resources is available from the corresponding author) while the implementation numerator was the number of completed projects.

Organizational maintenance was conceptualized with different metrics than implementation but remained consistent with the adoption conceptualization as we focused on SCI Action Canada’s partners. Organizational maintenance was then assessed by the extent to which partners remained engaged in the partnership over the years. For SCI Action Canada, the organizational maintenance numerator represented the number of partners that have remained partners since 2007. The organizational maintenance denominator for SCI Action Canada was the number of organizations that were partners at the launch of SCI Action Canada in 2007 (N = 16 partners).

Please see Table 1 for a summary of the description, numerators and denominators of reach, adoption, implementation and organizational maintenance.

**Table 1:** Overview of the descriptions, numerators and denominators of reach, adoption, implementation and organizational maintenance

**RE-AIM elements**	**Descriptions**	**Numerators**	**Denominators**
**Reach**			
*National*			
Total	The use of all reach data sources and includes adults with and without SCI that had contact with SCI Action Canada.	The addition of all numbers in the six data sources.	All Canadians with SCI.
End-user Intended and Direct	SCI and disability specific organizations who distributed SCI Action Canada resources to its audience and Canadians with SCI who had direct contact with SCI Action Canada.	The addition of all numbers in data sources 3 to 6.	All Canadians with SCI.
End-user Direct	Canadians with SCI who had direct contact with SCI Action Canada.	The addition of all numbers in data sources 4, 5, 6.	All Canadians with SCI.
*Regional*	Regional reach is specific to the two provinces that have community partners linked with SCI Action Canada, namely Alberta and Ontario.		
Total	The use of all reach data sources from Alberta and Ontario. It includes adults with and without SCI that had contact with SCI Action Canada.	The addition of Alberta and Ontario only numbers of the six data sources.	Canadians with SCI living in Alberta or Ontario.
End-user Intended and Direct	SCI and disability specific organizations in Alberta and Ontario who distributed SCI Action Canada resources to its audience and Canadians with SCI who had direct contact with SCI Action Canada.	The addition of Alberta and Ontario only numbers of data sources 3 to 6.	Canadians with SCI living in Alberta or Ontario.
End-user Direct	Canadians with SCI from Alberta and Ontario who had direct contact with SCI Action Canada.	The addition of Ontario and Alberta only numbers of data sources 4, 5, 6.	Canadians with SCI living in Ontario or Alberta.
*Engaged*	Canadians with SCI who were actively involved in SCI Action Canada research projects.	Data source 5: number of adults with SCI who participated in SCI Action projects.	Canadians with SCI who were contacted to participate in a SCI Action Canada research project.
**Adoption**			
*National*	The extent to which community organizations that provide service to Canadians with SCI are partners with SCI Action Canada.	Number of SCI Action Canada partners.	The total number of possible community organizations that provide service to Canadians with SCI.
*Regional*	The extent to which community organizations that provide service to Canadians with SCI living in Ontario and Alberta are partners with SCI Action Canada.	Number of SCI Action Canada partners.	The total number of possible community organizations that provide service to Canadians with SCI living in Ontario and Alberta.
*Engaged*	The extent to which community SCI Action Canada partners have adopted a SCI Action Canada program/initiative.	Number of SCI Action Canada partners that have adopted a SCI Action Canada program.	Number of SCI Action Canada partners.
**Implementation**	The extent to which SCI Action Canada has implemented its strategic plan.	Number of activities/projects in the strategic plan that are completed.	Total number of activities/projects outlined in SCI Action Canada’s strategic plan.
**Organizational Maintenance**	The extent to which SCI Action Canada’s partners remained partners.	Number of SCI Action Canada’s partners that remained partners.	Number of SCI Action Canada partners.

*Note*. SCI: Spinal cord injury. Please see the manuscript for the full descriptions of the data sources. Briefly, each data source consists of unique data (*e.g*., website users, number of resources given directly to adults with SCI) collected to calculate the numerators of reach.

### Effectiveness and individual maintenance

The effectiveness element is based on the main objective of the partnership: to increase population-level leisure time physical activity (LTPA) participation among adults with SCI. A five-year (data collected from 2007 to 2013) prospective cohort study of adults with SCI living in Ontario, Canada will be used to assess whether a difference exists in LTPA between the years prior to (<2010) and after the launch (2011 to 2013) of key SCI Action Canada programs, initiatives and resources (*e.g*., physical activity guidelines for adults with SCI [26], SCI Get Fit Toolkit [27]). Because data collection has only very recently been completed, no physical activity data are presented in this paper. Two physical activity analyses are planned for the effectiveness components. The first index of effectiveness is whether LTPA participation rates increased in the SCI population. Given that a 1% increase in physical activity in the general population has been estimated to save $15 million in healthcare costs [31], such an increase in activity would be deemed important and meaningful in our study, especially because approximately 50% of adults with SCI participate in zero minutes of LTPA per day [21]. Extrapolating at the population level, this 1% increase would represent over an additional 800 Canadians with SCI participating in LTPA. The second effectiveness index is a change in the proportion of Canadians with SCI meeting the Physical Activity Guidelines for Adults with SCI.

An additional effectiveness outcome is related to public awareness of SCI Action Canada and its programs/initiatives. This outcome index reflects the percentage of Canadians with SCI who are aware of: (a) SCI Action Canada, and/or (b) the Physical Activity Guidelines for adults with SCI. Questions to address these awareness outcomes have only recently been integrated into SCI Action Canada’s surveys and data are still being collected.

The maintenance evaluation of SCI Action Canada will solely focus on the LTPA data. The first goal of our evaluation is to determine if any increase in LTPA rates since the launch of our initiatives (2011) has been maintained at year five of the cohort study (2013). The second goal is to evaluate whether individuals with SCI who were meeting the guidelines after the launch of our initiatives are still meeting the guidelines at year five of the cohort study.

Note that all SCI Action Canada research projects were approved by the appropriate research ethics board (McMaster University, Queen’s University, or Western University) and informed consent was obtained from participants.

## Results

### Reach

#### National reach

Regarding total national reach, SCI Action Canada has indirectly reached 18% of Canadians with SCI. SCI Action Canada has an end-user indirect and direct national reach of 11% and a 3% end-user direct national reach.

#### Regional reach

SCI Action Canada has a total regional reach of 36% of Canadians with SCI living in Ontario or Alberta. Approximately 22% of Canadians with SCI have had intended or direct contact with SCI Action Canada (*i.e*., end-user indirect and direct regional reach). With respect to the end-user direct regional reach, 7% of Canadians with SCI living in Ontario or Alberta had direct contact with SCI Action Canada.

#### Engaged reach

Approximately 49% of the individuals who were contacted by SCI Action Canada have been directly involved with an SCI Action Canada research project.

### Adoption

The national adoption rate of SCI Action Canada was 46%, while the adoption ratio at the regional level was 76%. Of SCI Action Canada’s 16 partners, 33% of them adopted/implemented a program developed by SCI Action Canada (*i.e.,* engaged level adoption). Because SCI Action Canada had official partners in two Canadian provinces, the provincial level adoption rate was 15%.

### Implementation and organizational maintenance

SCI Action Canada implemented 92% of its planned programs, initiatives and resources, which included development and distribution of physical activity-promoting materials, research studies and knowledge mobilization projects. Regarding organizational maintenance, all 16 partners remain active in the partnership based upon regular participation on conference calls, attendance at meetings, and providing feedback on partnership goals. The result is 100% organizational maintenance as all of SCI Action Canada’s initial partners have remained partners.

## Discussion

The primary purpose of this paper was to demonstrate how to operationalize RE-AIM as an evaluation tool for large, multi-sectoral partnerships. Using the SCI Action Canada partnership as an example, we have demonstrated how to define and operationalize the RE-AIM dimensions and how to carry out a RE-AIM assessment of a multi-pronged and multi-sectoral partnership. As a secondary objective, the results of our RE-AIM analysis of SCI Action Canada provide data regarding the partnership’s impact at individual and organizational levels.

The SCI Action Canada example further illustrates RE-AIM’s potential to move beyond evaluation of single interventions or settings, to evaluation of multi-pronged and multi-sectoral partnership activities [15]. Demonstrating how RE-AIM can be used in university-community partnerships that move beyond randomized controlled trials and into ‘real world’ health promotion addresses a significant gap in the implementation science and RE-AIM literature [15]. Specifically, we have demonstrated that the RE-AIM framework can be applied to large national partnerships that engage both organizations and individuals. Given the increasing emphasis that funders and practitioners are placing on partnered approaches to health promotion [16,17], there is a need for tools to guide the evaluation of such approaches (*e.g*., RE-AIM,[1,4]). Our study has addressed this need by providing a RE-AIM evaluation template that can be used by partnerships, and data that other community-university partnerships can use to compare their rates of reach, adoption, implementation and organizational maintenance.

Our analysis has also demonstrated how partnerships can evaluate the outcomes of broad, multi-faceted activities that might not typically be taken into account if evaluation focuses only on specific, discrete activities/programs (*i.e*., the traditional use of the RE-AIM framework). For instance, SCI Action Canada materials (*e.g*., physical activity guidelines and toolkits) were distributed through a variety of partners and mechanisms; this was crucial for extending our reach. Had we not tracked distribution activities — and instead, only focused on evaluating the reach of discrete deliverable programs and initiatives, our total national reach would have only been significantly smaller. By including cross-cutting distribution activities, at the individual level, SCI Action Canada has a total national and regional reach of 18% and 36%, respectively, and direct national and regional reach of 3% and 7%, respectively. In comparison, Walk Kansas, a state-wide walking promotion, reached approximately 1% of the general population of Kansas [10]. Similarly, a weight-loss program in six counties in North Carolina reached an average of 1.2% of the population (range = 0.4% to 3%; [32]). Thus, by adopting a hybrid approach, based on pragmatics, in our RE-AIM evaluation, we were better able to quantify the impact of the partnership, and we can conclude that SCI Action Canada’s reach is just as good, if not better than other single program initiatives.

At an organizational level, SCI Action Canada has a high regional adoption ratio (76%), 92% implementation rate and perfect organizational maintenance. A strong partnership between university researchers and individuals in community-based organizations brings many benefits to the universities and communities (*e.g*., new approaches to tackle issues of interest; [33]). By developing SCI Action Canada research projects and knowledge mobilization activities based on the needs, expertise and resources of the community partners and university researchers [19], we have been able to ensure that our evidence-based tools and products are maximally beneficial to end-users. This approach has served to strengthen and maintain the commitment of all partners involved.

### Recommendations

Drawing on our experience evaluating SCI Action Canada and the limitations of our application of RE-AIM, we wanted to share the following recommendations for future partnerships planning an evaluation using the RE-AIM framework:

1. Use RE-AIM when creating the partnership’s strategic plan and designing projects and initiatives. Considering the RE-AIM dimensions during the planning stage will enable team members to design activities to collect necessary data from the onset and to prepare optimal data tracking procedures.

2. Be flexible when working with community partners and be prepared to ‘relax’ some of the scientific controls. For example, SCI Action Canada originally planned to compare provinces that did and did not have community partnership with SCI Action Canada. As a result, a decision was made not to add new ‘official’ partners as of 2010. However, in 2011, there was a national demand for SCI Action Canada programs/initiatives/resources. Given the overall goal of SCI Action Canada, the partnership decided to distribute the materials nationally. Since 2011, SCI Action Canada has added approximately 10 informal partners in Canada and distributed materials to four additional provinces. Because these organizations were not official partners, they were not included in the regional reach and adoption calculations.

3. Track all partnership activities, including research, presentations and distributed materials. Collect the most detailed data possible for each activity, including the number of end-users and intermediary persons that were invited and/or participated. Having this information would have allowed us to expand the engaged reach ratio beyond SCI Action Canada’s research projects.

4. We were unable to track the extent to which the same individuals with SCI were contacted on multiple occasions over time or participated in multiple programs/activities. We recommend that future partnerships try to document multiple contacts and program participation in the most pragmatic way possible. Collecting these data would provide more precise reach values, further enrich the data, and provide a temporal component. Such data could also be useful for community partners as they could get a sense of whether their organization is reaching new end-users.

5. Identify the main outcomes of interest for the partnership (*e.g*., increased awareness, change in attitudes) at the onset and incorporate measures of these outcomes across all research projects, interventions or program evaluations that are conducted by partnership members. This suggestion is especially important if you have researchers from different universities collaborating under the umbrella of the partnership.

## Conclusion

SCI Action Canada’s programs, initiatives and resources are reaching widely across Canada, and the partnership has maintained strong relationships with community and research partners. These data suggest that SCI Action Canada is on the right track to achieving its mission to inform, teach and enable Canadians with SCI to become more physically active. These conclusions were derived by extending and operationalizing the RE-AIM framework to evaluate the multi-sectoral activities undertaken by the partnership. As such, this operationalization of RE-AIM is transferable to other settings, and we encourage the use of RE-AIM to evaluate the public health impact of other large, multi-sectoral partnerships.

## Competing interests

The authors do not have any competing interests to declare.

## Authors’ contributions

All authors contributed in conceptualizing, writing and editing of this manuscript. SNS lead the data acquisition and analysis. KMG and ALC are SCI Action Canada researchers that contributed their knowledge of the partnership to the manuscript. PE provided expertise pertaining to RE-AIM. All authors approved the manuscript.

## References

[B1] Glasgow RE Vogt TM Boles SM Evaluating the public health impact of health promotion interventions: the RE-AIM framework Am J Public Health 1999 89 1322 1327 10.2105/AJPH.89.9.1322 10474547 PMC1508772

[B2] Glasgow RE McKay HG Piette JD Reynolds KD The RE-AIM framework for evaluating interventions: what can it tell us about approaches to chronic illness management? Patient Educ Couns 2001 44 119 127 10.1016/S0738-3991(00)00186-5 11479052

[B3] Dzewaltowski DA Estabrooks PA Glasgow RE The future of physical activity behavior change research: what is needed to improve translation of research into health promotion practice? Exerc Sport Sci Rev 2004 32 57 63 10.1097/00003677-200404000-00004 15064649

[B4] Kessler RS Purcell EP Glasgow RE Klesges LM Benkeser RM Peek CJ What does it man to “employ” the RE-AIM model? Eval Health Prof 2013 36 67 72 10.1177/0163278712460546 22615498

[B5] Akers JD Estabrooks PA Davy BM Translational research: bridging the gap between long-term weight loss maintenance research and practice J Am Diet Assoc 2010 110 1511 1522 10.1016/j.jada.2010.07.005 20869490 PMC2967429

[B6] Kahwati LC Lance TX Jones KR Kinsinger LS RE-AIM evaluation of the veterans health Administration’s MOVE! weight management program Transl Behav Med 2011 1 551 560 10.1007/s13142-011-0077-4 24073079 PMC3717682

[B7] Dunton GF Lagloire R Robertson T Using the RE-AIM framework to evaluate the statewide dissemination of a school-based physical activity and nutrition curriculum: “Exercise Your Options” Am J Health Promot 2009 23 229 232 10.4278/ajhp.071211129 19288843 PMC2657926

[B8] Finch C Li G, Baker SP Implementing and Evaluating Interventions Injury research: theories, methods, and approaches 2012 New York: Springer US 619 639

[B9] Li F Harmer P Glasgow R Mack KA Sleet D Fisher KJ Kohn MA Millet LM Mead J Xu J Lin ML Yang T Sutton B Tompkins Y Translation of an effective Tai Chi intervention into a community-based falls-prevention program Am J Public Health 2008 98 1195 1198 10.2105/AJPH.2007.120402 18511723 PMC2424086

[B10] Estabrooks PA Bradshaw M Dzewaltowski DA Smith-Ray RL Determining the impact of Walk Kansas: applying a team-building approach to community physical activity promotion Ann Behav Med 2008 36 1 12 10.1007/s12160-008-9040-0 18607666

[B11] Gaglio B Shoup J Glasgow RE The RE-AIM framework: a systematic review of use over time Am J Public Health 2013 103 e38 e46 23597377 10.2105/AJPH.2013.301299 PMC3698732

[B12] Payne JM France KE Henley N D’Antoine HA Bartu AE O’Leary CM Elliott EJ Bower C Geelhoed E RE-AIM evaluation of the alcohol and pregnancy project: educational resources to inform health professionals about prenatal alcohol exposure and fetal alcohol spectrum disorder Eval Health Prof 2011 34 57 80 10.1177/0163278710381261 21292723

[B13] Vick L Duffy SA Ewing LA Rugen K Zak C Implementation of an inpatient smoking cessation programme in a veterans affairs facility J Clin Nurs 2013 22 866 880 22882776 10.1111/j.1365-2702.2012.04188.x

[B14] Finch CF Gabbe BJ Lloyd DG Cook J Young W Nicholson M Seward H Donaldson A Doyle TLA Towards a national sports safety strategy: addressing facilitators and barriers towards safety guideline uptake Inj Prev 2011 17 1 10 21343627 10.1136/ip.2010.031385

[B15] Finch CF Donaldson A A sports setting matrix for understanding the implementation context for community sport Br J Sports Med 2010 44 973 978 10.1136/bjsm.2008.056069 19201766

[B16] Hall K Vogel A Stipelman B Stokols D Morgan G Gehlert S A four-phase model of transdisciplinary team-based research: goals, team processes, and strategies Transl Behav Med 2012 2 415 430 10.1007/s13142-012-0167-y 23483588 PMC3589144

[B17] Wanless D Jones N Anderson R Bowley F Gupta S Hall J Lawson R Naidoo B Roberts M Sabharwal H Skinner J Stevenson L Stone I Sweatman H Secruing good health for the whole population 2004 Norwich: HM Treasury

[B18] Day L Finch CF Hill KD Haines TP Clemson L Thomas M Thompson C A protocol for evidence-based targeting and evaluation of statewide strategies for preventing falls among community-dwelling older people in Victoria, Australia Inj Prev 2011 17 e3 10.1136/ip.2010.030775 21186224 PMC3064867

[B19] Martin Ginis KA Latimer-Cheung A Corkum S Ginis S Anathasopoulos P Arbour-Nicitopoulos K Gainforth H A case study of a community-university multidisciplinary partnership approach to increasing physical activity participation among people with spinal cord injury Transl Behav Med 2012 2 516 522 10.1007/s13142-012-0157-0 24073152 PMC3717928

[B20] Groah SL Nash MS Ward EA Libin A Mendez AJ Burns P Elrod M Hamm LF Cardiometabolic risk in community-dwelling persons with chronic spinal cord injury J Cardiopulm Rehabil Prev 2011 31 73 80 10.1097/HCR.0b013e3181f68aba 21045711

[B21] Martin Ginis KA Latimer AE Arbour-Nicitopoulos KP Buchholz AC Bray SR Craven BC Hayes KC Hicks AL McColl MA Potter PJ Smith K Wolfe DL Leisure time physical activity in a population-based sample of people with spinal cord injury part I: demographic and injury-related correlates Arch Phys Med Rehabil 2010 91 722 728 10.1016/j.apmr.2009.12.027 20434609

[B22] Mercer SL DeVinney BJ Fine LJ Green LW Dougherty D Study designs for effectiveness and translation research: identifying trade-offs Am J Prev Med 2007 33 139 154 e132 10.1016/j.amepre.2007.04.005 17673103

[B23] Glasgow RE What does it mean to be pragmatic? Pragmatic methods, measures, and models to facilitate research translation Health Educ Behav 2013 40 257 265 10.1177/1090198113486805 23709579

[B24] Oliver M An introduction to the evaluation of learning technology Edu Technol Soc 2000 3 20 30

[B25] Graham ID Logan J Harrison MB Straus SE Tetroe J Caswell W Robinson N Lost in knowledge translation: time for a map? J Contin Educ Health 2006 26 13 24 10.1002/chp.47 16557505

[B26] Martin Ginis KA Hicks AL Latimer AE Warburton DER Bourne C Ditor DS Goodwin DL Hayes KC McCartney N McIlraith A Pomerleau P Smith K Stone JA Wolfe DL The development of evidence-informed physical activity guidelines for adults with spinal cord injury Spinal Cord 2011 49 1088 1096 10.1038/sc.2011.63 21647164

[B27] Arbour-Nicitopoulos KP Martin Ginis KA Latimer-Cheung AE Bourne C Campbell D Cappe S Ginis S Hicks AL Pomerleau P Smith K Development of an evidence-informed leisure time physical activity resource for adults with spinal cord injury: the SCI Get Fit Toolkit Spinal Cord 2013 51 491 500 10.1038/sc.2013.7 23608809

[B28] Latimer-Cheung AE Arbour-Nicitopoulos KP Brawley LR Gray C Wilson AJ Prapavessis H Tomasone JR Wolfe DL Martin Ginis KA Developing physical activity interventions for adults with spinal cord injury. Part 2: motivational counseling and peer-mediated interventions for people intending to be active Rehabil Psychol 2013 58 307 315 23978086 10.1037/a0032816

[B29] Gainforth HL Latimer-Cheung AE Athanasopoulos P Martin Ginis KA Examining the effectiveness of a knowledge mobilization initiative for disseminating the physical activity guidelines for people with spinal cord injury Disabil Health J 2013 6 260 265 10.1016/j.dhjo.2013.01.012 23769486

[B30] Farry A Baxter D The incidence and prevalence of spinal cord injury in Canada 2010 Canada: Rick Hansen Institute & Urban Features

[B31] Katzmarzyk P Gledhill N Shephard R The economic burden of physical activity in Canada CMAJ 2000 163 1435 1440 11192648 PMC80410

[B32] Samuel-Hodge CD Ba G Johnston LF Kraschnewski JL Aa G Norwood AF Glasgow RE Gold AD Graham JW Evenson KR Stearns SC Gizlice Z Keyserling TC Rationale, design, and sample characteristics of a practical randomized trial to assess a weight loss intervention for low-income women: the Weight-Wise II Program Contemp Clin Trials 2012 33 93 103 10.1016/j.cct.2011.08.009 21930244

[B33] Buys N Bursnall S Establishing university–community partnerships: processes and benefits J Higher Educ Pol Manage 2007 29 73 86 10.1080/13600800601175797

